# Identification of Diagnostic Schizophrenia Biomarkers Based on the Assessment of Immune and Systemic Inflammation Parameters Using Machine Learning Modeling

**DOI:** 10.17691/stm2023.15.6.01

**Published:** 2023-12-27

**Authors:** I.K. Malashenkova, S.A. Krynskiy, D.P. Ogurtsov, N.A. Khailov, P.V. Druzhinina, A.V. Bernstein, A.V. Artemov, G.Sh. Mamedova, N.V. Zakharova, G.P. Kostyuk, V.L. Ushakov, M.G. Sharaev

**Affiliations:** MD, PhD, Head of the Laboratory of Molecular Immunology and Virology; National Research Center “Kurchatov Institute”, 1 Akademika Kurchatova Square, Moscow, 123182, Russia; Senior Researcher, Laboratory of Clinical Immunology; Federal Research and Clinical Center of Physical-Chemical Medicine, Federal Medical Biological Agency of Russia, 1A Malaya Pirogovskaya St., Moscow, 119435, Russia; MD, PhD, Senior Researcher, Laboratory of Molecular Immunology and Virology; National Research Center “Kurchatov Institute”, 1 Akademika Kurchatova Square, Moscow, 123182, Russia; Junior Researcher, Department of Mental Disorders in Neurodegenerative Brain Diseases; N.A. Alekseyev Psychiatric Clinical Hospital No.1, 2 Zagorodnoye Shosse, Moscow, 117152, Russia; MD, PhD, Senior Researcher, Laboratory of Molecular Immunology and Virology; National Research Center “Kurchatov Institute”, 1 Akademika Kurchatova Square, Moscow, 123182, Russia; Researcher, Laboratory of Clinical Immunology; Federal Research and Clinical Center of Physical-Chemical Medicine, Federal Medical Biological Agency of Russia, 1A Malaya Pirogovskaya St., Moscow, 119435, Russia; MD, PhD, Senior Researcher, Resource Center of Molecular and Cell Biology; National Research Center “Kurchatov Institute”, 1 Akademika Kurchatova Square, Moscow, 123182, Russia; PhD Student; Skolkovo Institute of Science and Technology (Skoltech), Territory of Skolkovo Innovation Center, Bldg. 1, 30 Bolshoy Boulevard, Moscow, 121205, Russia; Intern Researcher, Biomedically Informed Artificial Intelligence Laboratory (BIMAI-lab); The University of Sharjah, the United Arab Emirates, Sharjah, 27272; DSc, Professor; Skolkovo Institute of Science and Technology (Skoltech), Territory of Skolkovo Innovation Center, Bldg. 1, 30 Bolshoy Boulevard, Moscow, 121205, Russia; PhD, Senior Researcher; Skolkovo Institute of Science and Technology (Skoltech), Territory of Skolkovo Innovation Center, Bldg. 1, 30 Bolshoy Boulevard, Moscow, 121205, Russia; Junior Researcher, Laboratory of Fundamental Research Methods; N.A. Alekseyev Psychiatric Clinical Hospital No.1, 2 Zagorodnoye Shosse, Moscow, 117152, Russia; MD, PhD, Head of the Laboratory of Fundamental Research Methods; N.A. Alekseyev Psychiatric Clinical Hospital No.1, 2 Zagorodnoye Shosse, Moscow, 117152, Russia; MD, DSc, Professor, Head Physician; N.A. Alekseyev Psychiatric Clinical Hospital No.1, 2 Zagorodnoye Shosse, Moscow, 117152, Russia; PhD, Associate Professor, Leading Researcher; Insitute of Perspective Brain Research, M.V. Lomonosov Moscow State University, Bldg. 1, 27 Lomonosov Avenue, Moscow, 119192, Russia; Senior Researcher, Department of Mental Disorders in Neurodegenerative Brain Diseaseas; N.A. Alekseyev Psychiatric Clinical Hospital No.1, 2 Zagorodnoye Shosse, Moscow, 117152, Russia; Senior Researcher; National Research Nuclear University MEPhI, 31 Kashirskoye Shosse, Moscow, 115409, Russia; Senior Teacher; Skolkovo Institute of Science and Technology (Skoltech), Territory of Skolkovo Innovation Center, Bldg. 1, 30 Bolshoy Boulevard, Moscow, 121205, Russia; Head of the Biomedically Informed Artificial Intelligence Laboratory (BIMAI-lab); The University of Sharjah, the United Arab Emirates, Sharjah, 27272; Senior Researcher, Department of Mental Disorders in Neurodegenerative Brain Diseases; N.A. Alekseyev Psychiatric Clinical Hospital No.1, 2 Zagorodnoye Shosse, Moscow, 117152, Russia

**Keywords:** schizophrenia, systemic inflammation, diagnostic biomarkers, immunity, machine learning, cytokines

## Abstract

Disorders of systemic immunity and immune processes in the brain have now been shown to play an essential role in the development and progression of schizophrenia. Nevertheless, only a few works were devoted to the study of some immune parameters to objectify the diagnosis by means of machine learning. At the same time, machine learning methods have not yet been applied to a set of data fully reflecting systemic characteristics of the immune status (parameters of adaptive immunity, the level of inflammatory markers, the content of major cytokines). Considering a complex nature of immune system disorders in schizophrenia, incorporation of a broad panel of immunological data into machine learning models is promising for improving classification accuracy and identifying the parameters reflecting the immune disorders typical for the majority of patients.

**The aim of the study** is to assess the possibility of using immunological parameters to objectify the diagnosis of schizophrenia applying machine learning models.

**Materials and Methods:**

We have analyzed 17 immunological parameters in 63 schizophrenia patients and 36 healthy volunteers. The parameters of humoral immunity, systemic level of the key cytokines of adaptive immunity, anti-inflammatory and pro-inflammatory cytokines, and other inflammatory markers were determined by enzyme immunoassay. Applied methods of machine learning covered the main group of approaches to supervised learning such as linear models (logistic regression), quadratic discriminant analysis (QDA), support vector machine (linear SVM, RBF SVM), k-nearest neighbors algorithm, Gaussian processes, naive Bayes classifier, decision trees, and ensemble models (AdaBoost, random forest, XGBoost). The importance of features for prediction from the best fold has been analyzed for the machine learning methods, which demonstrated the best quality. The most significant features were selected using 70% quantile threshold.

**Results:**

The AdaBoost ensemble model with ROC AUC of 0.71±0.15 and average accuracy (ACC) of 0.78±0.11 has demonstrated the best quality on a 10-fold cross validation test sample. Within the frameworks of the present investigation, the AdaBoost model has shown a good quality of classification between the patients with schizophrenia and healthy volunteers (ROC AUC over 0.70) at a high stability of the results (σ less than 0.2). The most important immunological parameters have been established for differentiation between the patients and healthy volunteers: the level of some systemic inflammatory markers, activation of humoral immunity, pro-inflammatory cytokines, immunoregulatory cytokines and proteins, Th1 and Th2 immunity cytokines. It was for the first time that the possibility of differentiating schizophrenia patients from healthy volunteers was shown with the accuracy of more than 70% with the help of machine learning using only immune parameters.

The results of this investigation confirm a high importance of the immune system in the pathogenesis of schizophrenia.

## Introduction

Schizophrenia is a severe chronic psychiatric disease with cognitive function impairment, emotional and psychomotor disorders [1]. A high rate of disability and reduced life longevity is often observed in schizophrenia [2]. Early diagnosis and timely administered therapy are necessary to reduce the risk of unfavorable course of the disease. An important direction of researches aimed to improve schizophrenia diagnosing is the development of approaches to the objectification of the diagnosis using machine learning (ML) models based on the clinically significant biomarkers [3, 4]. In clinical practice, biomarkers are important for determining several factors: diagnosis clarification, verification of the disease stage, selection of the optimal treatment plan, long-term prognosis of the disease. However, the clinical significance (determined by sensitivity, specificity, prognostic value, etc.) has not been established at the relevant levels of evidence for the majority of the presently found schizophrenia biomarkers, and therefore, no provision is made for the application of biomarkers in the existing clinical recommendations [5].

A large volume, high dimensionality, and heterogeneity of multimodal laboratory data make it difficult to integrate all available modalities within the framework of a single study, therefore, interest is growing among the clinicians in the current approaches to the integration of heterogeneous data, ML and deep learning (DL) methods [6].

Presently, it has been proved that systemic immunity disorders and immune processes in the central nervous system (CNS) play an essential role in the development and progression of schizophrenia [7–9]. Nevertheless, there are a few works devoted to the study of some immunity parameters for objectifying the diagnosis of schizophrenia with ML methods [10]. Besides, the ML methods have not yet been applied to the datasets fully reflecting systemic characteristics of immune status (parameters of adaptive immunity, the level of inflammatory markers, content of major cytokines) [11–13]. Taking into consideration a complex character of immune system disorders in schizophrenia, inclusion into ML a broad panel of immunological data is a promising task for improving classification accuracy and selecting the variable parameters typical for the majority of patients. From the practical point of view, the results of data analysis by ML method may be used for the development of a new panel of markers having a diagnostic/prognostic value and become a basis for creating a prototype of a clinical decision support system using the selected features.

**The aim of the study** is to assess the possibility of using immunological parameters to objectify the diagnosis of schizophrenia applying machine learning models.

## Materials and Methods

The clinical data presented in the given work are part of the research program “Molecular and neurophysiological markers of endogenous diseases” carried out at the N.A. Alekseyev Psychiatric Clinical Hospital No.1 of the Moscow Health Department (Russia) and approved by the Independent Interdisciplinary Committee on the ethical expertise of clinical researches (Protocol No.12 of July 14, 2017). Some results of this project have been published previously [14–16].

### Patients

The study included 63 patients (36 men and 27 women, average age 29±3 years) hospitalized at the N.A. Alekseyev Psychiatric Clinical Hospital No.1 with the diagnosis of schizophrenia spectrum disorders (F20 and F25 according to ICD-10). All participants gave written informed consent after a full description of the investigation procedures in compliance with the Declaration of Helsinki.

Patients were selected according to the following inclusion criteria: patient’s state must meet the criteria of schizophrenia presented in ICD-10 and DSM-5 (Diagnostic and Statistical Manual of Mental Disorders, fifth edition); insight into the ill condition; preserving the memory of psychotic symptoms; informed consent for participation in the study.

Exclusion criteria were as follows: schizoaffective and affective disorders; organic brain diseases; severe somatic and/or neurological conditions potentially affecting physiology or structure of the brain; signs of psychoactive substance abuse; acute chronic somatic and infectious inflammatory diseases or their exacerbation.

The control group consisted of 36 unrelated volunteers comparable in age and gender with the patients (21 men and 15 women, average age — 30±2 years). The volunteers and patients with schizophrenia were examined according to the same protocol.

***Study design:*** cross-sectional, observational, case–control. Patients’ condition was diagnosed for two days. Before biomaterial collection, a clinical picture of psychosis was determined and objective information was clarified (from relatives or medical cards). The day prior to biomaterial collection, the condition was psychometrically assessed using the positive and negative syndrome scale for schizophrenia (PANSS) [17] (Table 1). Clinical investigation was performed by two experienced psychiatrists who collected all necessary data: questioning the relatives, analysis of medical cards, results of physical and laboratory tests, etc. The mental state at the time of scanning was characterized by criticism towards the previous psychosis and preserved memory with the ability to present subjective history of disease development (with a detailed content of delusion and assessment of affective condition in that period).

**T a b l e 1:** Characteristics of patients with schizophrenia (n=63)

Parameters	Patients (M±σ)
Average age (years)	28.7±7.7
Average age of disease onset (years)	18.0±4.2
Average age of disease manifestation (years)	21.3±4.0
Disease duration from prodrome (years)	9.6±7.8
Disease duration from manifestation (years)	6.5±6.0
PANSS general (points)	94.1±17.1
PANSS P (points):	17.3±6.4
P1 (delusion)	2.9±1.2
P2 (thought disorder)	3.1±1.3
P3 (hallucinations)	2.5±1.8
P4 (excitation)	1.7±1.1
P5 (ideas of grandeur)	1.5±0.8
P6 (suspicion)	3.5±1.4
P7 (hostility)	2.2±1.1
PANSS N (points)	27.5±7.4
PANSS G (points)	49.3±8.7
NSA-4 (points)	20.4±4.6

N o t e s: NSA-4 — negative symptom assessment scale; PANSS — positive and negative syndrome scale; PANSS P — severity of productive symptoms; PANSS N — severity of negative symptoms; PANSS G — intensity of other psychotic disorders according to the general psychological scale.

### Immunological investigations

Seventeen immunological indicators have been analyzed. They included parameters of humoral immunity (IgA, IgM, and IgG); systemic level of the key pro-inflammatory and anti-inflammatory cytokines, and other inflammatory markers (C-reactive protein (CRP), cortisol, circulating immune complexes (CIC)), brain-derived neurotrophic factor (BDNF) identified by enzyme immunoassay using reagent kits manufactured by CHEMA LLC (Russia), Cytokine LLC (Russia), R&D Systems (USA).

### Statistical data processing

The results were statistically processed using Python 3.9.0 software (Python Software Foundation) in the form of NumPy, Pandas, SkLearn libraries, which are used in ML.

In the present study, quantitative continuous variables have been analyzed. The analysis of variables in dynamics was not performed since the study was crosssectional. Two independent samples were examined: patients with schizophrenia and healthy volunteers.

As part of the initial data analysis, the distribution normality was determined using Shapiro–Wilk test. Clinical data were presented in the form of means with a standard deviation (M±σ), immunological parameters as medians and 25^th^ and 75^th^ percentiles (Me [25; 75]).

Such metrics as accuracy (ACC) and area under the ROC curve (ROC AUC) were used to evaluate the quality of classification. Accuracy measures the proportion of correctly predicted values in relation to the total number of responses, and it is higher for the models giving more correct prognoses. However, for the datasets with heterogeneous class representation, accuracy may be non-optimal metrics. ROC AUC measures an area under the ROC curve reflecting the ability of the model to discriminate objects belonging to two classes at different classification thresholds. ROC AUC values lie in the range from 1 to 0, the higher value indicating the better model performance. Since the number of patients with schizophrenia and healthy volunteers was different, ROC AUC was used as the main metric of model quality in the present study. A threshold level of ROC AUC was 0.70, which means a good classification quality.

To assess the stability of the obtained models for each of the ACC and ROC AUC metrics in all folds, a standard deviation (σ) was calculated. A lower value of σ denoted a more stable model work, with σ less than 0.2 being considered small, and from 0.2 to 0.5 moderate. Within the frameworks of this study, models having σ for ACC and ROC AUC less than 0.2 were considered stable.

### Machine learning methods

The ML methods applied covered the main groups of approaches to a supervised learning such as linear models (logistic regression), quadratic discriminant analysis (QDA), support vector machine (linear SVM, RBF SVM), k-nearest neighbors algorithm, Gaussian processes, naive Bayes classifier, decision trees, and ensemble models (AdaBoost, random forest, XGBoost). These methods comprise a wide spectrum of approaches in ML, each having its strong points. Consideration of various approach groups allowed us to establish, which of them copes best with the given task. Speaking on the family of the ensemble approaches in more detail, it should be noted that they are based on the idea of combining several classifiers in a single strong one. For example, each weak classifier in AdaBoost (adaptive boosting) may be any learning algorithm (taking the values of negative and positive weights). At each learning iteration, AdaBoost increases the weights of the incorrectly classified samples in order to focus the next classifier on those samples where the previous classifiers have made mistakes. After training, all weak classifiers are integrated using weighted voting, where each classifier is assigned a weight based on its accuracy during classification, which makes it possible to obtain a strong classifier representing an ensemble of weak classifiers. When a final strong classifier is used for categorization of new samples, it takes into account the vote of all weak classifiers according to their weights, achieving thereby a more accurate and stable classification.

For each ML model, it is possible to evaluate relevance or contribution of various features in relation to prediction, i.e. to assess qualitatively the feature importance in the process of decision-making by the model. Feature importance may be computed by different methods such as replacement importance, Gini impurity importance, or importance coefficient value, which allows for determining the effect of each separate variable on the predicted variable and assessing its role in modeling. The result of calculating the importance coefficients may be presented as a table, enabling one to analyze the significance of each variable for modeling. Higher values indicate greater importance. Although feature importance may give knowledge on the relative contribution of each, this parameter should be interpreted with caution. It is not always implying cause-and-effect relations or direct relations between the features. Moreover, the interpretation of function importance depends on the specific model and context, and it should not be considered along with other assessment measures and knowledge in the subject area for comprehensive understanding. Thus, using the parameter of function importance, it is possible to evaluate function relevance in the ML model. This parameter provides interpretability, although it should be used as part of a wider analysis rather than a single basis for making conclusions.

The training process and model quality assessment were performed using a 10-fold StratifiedKFold crossvalidation. As a result, 10 models for each ML method have been created, the quality of each model was determined on a test sample (20% of data). In this way, we obtained a sample of ten values for each quality metric for the selected set of hyperparameters. Next, a mean and standard deviation were found for each quality metric. For ML methods, which demonstrated the best quality, feature importance was analyzed for the prediction obtained from the best fold. The most significant features fitting the 70% quantile threshold were selected.

## Results

During the experiments, it has been found that the best quality in the test sample on the 10 folds was demonstrated by the ensemble AdaBoost model with the area under the ROC curve of 0.71±0.15 and an average accuracy (ACC) of 0.78±0.11. Within the scope of this study, the AdaBoost model showed a good quality of classification between the patients with schizophrenia and healthy volunteers (ROC AUC more than 0.70) at a high stability of the results (σ less than 0.2) (Table 2).

**T a b l e 2:** Results of objectifying the diagnosis of schizophrenia using machine learning models

Experiment No.	Methods	Binary classification	
		Mean ROC AUC (σ)	Mean ACC (σ)
1	**AdaBoost**	**0.71 (0.15)**	**0.78 (0.11)**
2	Decision tree	0.56 (0.25)	0.60 (0.21)
3	Gaussian process	0.52 (0.12)	0.66 (0.08)
4	Linear SVM	0.49 (0.03)	0.67 (0.05)
5	Logistic regression	0.62 (0.21)	0.70 (0.16)
6	Naive Bayes	0.58 (0.20)	0.54 (0.19)
7	k-nearest neighbors	0.63 (0.22)	0.67 (0.18)
8	Neural networks	0.58 (0.14)	0.67 (0.09)
9	Quadratic discriminant analysis	0.62 (0.15)	0.70 (0.13)
10	RBF SVM	0.50 (0.0)	0.68 (0.03)
11	Random forest	0.54 (0.17)	0.67 (0.12)
12	XGBoost classifier	0.68 (0.21)	0.75 (0.19)

N o t e s: ROC AUC and ACC metrics are computed as a mean across a test data sample using a 10-fold cross-validation; the most accurate results of classification are marked in bold.

The results of the feature analysis are represented in Table 3 in order of importance for the quality of classification using the AdaBoost model.

**T a b l e 3:** Feature importance ranking for the AdaBoost model with a 70% quantile threshold — 0.084

No.	Features	Importance coefficient
1	IL-8	0.12
2	CIC	0.1
3	IL-4	0.1
4	IFN-γ	0.1
5	TNF-α	0.1
6	Cortisol	0.08

N o t e s: IFN-γ — interferon-γ, IL — interleukin, TNF-α — tumor necrosis factor α, CIC — circulating immune complexes.

Table 4 shows feature importance ranking using XGBoost classifier, having the second values of ROC AUC and ACC, high or moderate stability of the classification results (ROC AUC — 0.68±0.21; ACC — 0.75±0.19).

**T a b l e 4:** Feature importance ranking for the XGBoost classifier model with a 70% quantile threshold — 0.081

No.	Features	Importance coefficient
1	IL-1β	0.128
2	IL-10	0.1
3	Cortisol	0.089
4	CIC	0.088
5	TNF-α	0.088

N o t e s: IL — interleukin, TNF-α — tumor necrosis factor α, CIC — circulating immune complexes.

To make the obtained data more clear, we also presented feature importance ranking using logistic regression classification model, which is more simple and illustrative relative to the AdaBoost and XGBoost classifiers and has the third-largest value of ROC AUC with high or moderate stability of the results (ROC AUC — 0.62±0.21, ACC — 0.70±0.16) (see Table 2; Table 5).

**T a b l e 5:** Feature importance ranking for the logistic regression model with 70% quantile threshold — 0.303

No.	Features	Importance coefficient
1	IL-1RA	1.208
2	IFN-γ	0.889
3	CIC	0.678
4	CRP	0.574
5	IgM	0.336

N o t e s: CRP — C-reactive protein, Ig — immunoglobulin, IFN-γ — interferon-γ, IL-1RA — interleukin 1β receptor antagonist, CIC — circulating immune complexes.

Thus, ranking by the importances of features relevant for the three models with the greatest predictive power allowed us to establish parameters which were most significant for discrimination between the patients with schizophrenia and healthy volunteers.

## Discussion

As a result of our investigation, it was shown for the first time that it is possible to diagnose schizophrenia with the accuracy greater than 70% using machine learning models built only on a set of indicators of immune system condition. The given level of diagnostic accuracy was achieved despite the fact that at this stage we did not intend to include the results of clinical examination into the machine learning models, in contrast to the work [10], where both separate immunological data and the results of neuropsychological testing were used.

The study has revealed for the first time a complex of parameters of natural and adaptive immunity which are most important for classification of patients and individuals without psychiatric diseases: the level of some markers of systemic inflammation and activation of humoral immunity (CRP, cortisol, CIC), pro-inflammatory cytokines (IL-1β, IL-8, TNF-α), immunoregulatory proteins (IL-1RA), Th1 immunity cytokines (IFN-γ), Th2 cytokines (IL-4), and immunoregulatory cytokines (IL-10).

An important role of systemic inflammation parameters and the level of pro-inflammatory cytokines is worth mentioning. The literature and our data confirm the fact that patients with schizophrenia show the signs of systemic inflammation, which are most intensive in the first episode of the disease and in exacerbations. In disease exacerbation, the levels of pro-inflammatory cytokines IL-8, TNF-α, and CRP, an acute-phase protein, increase [18–20]. A high CRP level has been also shown to be associated with a more severe course of psychosis in schizophrenia and subsequent cognitive function decline [18, 21]. A definite level of immunoinflammatory activation, according to our data, remains also in the period of medically induced remission. However, application of ML methods has not yet been studied for verification of schizophrenia diagnosis by assessing the level of systemic inflammation markers [11, 12]. At the same time, there are several investigations, in which interconnections have been established between the level of some markers of systemic inflammation and clinical characteristics of schizophrenia including the intensity of cognitive function impairment (TNF-α, IL-2, IL-6, IL-8, cortisol), neurocognitive defect (IL-1β, sIL-1RA, TNF-α), acute or chronic course of the disease (TNF-α, IL-2, IL-6, IL-8, cortisol) [22, 23].

Cytokines of innate immunity, IL-1β, IL-8, TNF-α, play an important role in triggering and maintaining the systemic inflammatory response, pyrogenic reactions, and functions of adaptive immunity [24]. The elevation of their level in the CNS, which may be observed, for example, in neuroinfections, autoimmune diseases, mental diseases, causes a damaging effect on neurons and glial cells, facilitates neurodegeneration (see, for example, review [25]). According to the data of the metaanalysis [18], the level of IL-6, TNF-α cytokines, and the receptor antagonist IL-1RA is increased in acute schizophrenia. The levels of IL-1β and IL-6 are also elevated in chronic schizophrenia, as shown in the same meta-analysis. According to our data, the increased IL-8 level is noted in patients with schizophrenia irrespective of the intensity of clinical symptoms [26].

IFN-γ is an important cytokine activating functions of CD3+CD4+ T helpers type 1, CD3+CD8+ cytotoxic T cells, and CD3–CD16+CD56+ NK cells, which provide antiviral defense, and stimulating presentation of antigens to lymphocytes by the cells of the innate immunity. In inflammatory conditions, IFN-γ is actively produced by T lymphocytes and NK cells. Our results and the literature data show that its content increases in schizophrenia reflecting complex activation of mechanisms of adaptive and innate immunity in patients [26, 27]. In the frameworks of this study, IFN-γ has been shown to be one of the most significant indicators for confirming the diagnosis of schizophrenia with the help of machine learning models.

Immunological parameters reflecting activation of Th2 cytokines and immunoregulatory mechanisms such as the level of IL-4 cytokine, IL-1RA immunoregulatory protein, and CIC are also referred to the number of diagnostically significant markers identified in our investigation. According to the literature data, activation of the innate (natural) immunity response in patients with schizophrenia is followed by the activation of Th2 cells of adaptive immunity. The signs of activation of Th2 immune response in schizophrenia include the increase of the IL-4 and IL-10 levels in blood serum, decrease of Th1/Th2 cytokine level ratio (IFN-γ/IL-4, IFN-γ/IL-10, IL-2/IL-4, TNF-α/IL-4) [28]. De Campos-Carli et al. [29] have detected association between the intensity of cognitive disorders and elevated levels of Th2 cytokine IL-33 in the patients’ blood [29]. Activation of Th2 response in patients with schizophrenia may participate in the impairment of neurotransmitter exchange in the CNS involved in the pathogenesis of the negative symptoms in schizophrenia [30]. Previously, we have established for the first time that the immunological profile, which is characterized by the increase of IL-10 content and moderate signs of systemic inflammation, is associated with marked negative symptoms in patients with schizophrenia [26].

Thus, the revealed complex of immunological indicators (IL-8, CIC, IL-4, IFN-γ, TNF-α, cortisol) is significant for diagnosing schizophrenia. It should be noted that these parameters are associated with important clinical characteristics of the disease. The literature data indicate their participation in the schizophrenia pathogenesis.

Identification of these immunoinflammatory biomarkers suggests less time, economic, and organizational expenses than neuroimaging and spinal fluid examination. Our study has shown that the application of immunological parameters in clinical practice is a promising area for objectifying the diagnosis of schizophrenia.

## Conclusion

The results of this study have shown for the first time that machine learning methods and the assessment of only the parameters of systemic inflammation, innate and adaptive immunity provide the possibility to validate the diagnosis of schizophrenia with the accuracy greater than 70%. Our results confirm the pathogenetic significance of immune system state and immunoinflammatory syndrome in schizophrenia.

At the next stage of the research, we are going to analyze a wide spectrum of immunological parameters as part of multimodal data using machine learning methods in order to create interpretable models which might be used to detect clinical and immunological variants of psychoses.
